# Systemic Prenatal Stress Exposure through Corticosterone Application Adversely Affects Avian Embryonic Skin Development

**DOI:** 10.3390/biology12050656

**Published:** 2023-04-26

**Authors:** Morris Gellisch, Martin Bablok, Satya Srirama Karthik Divvela, Gabriela Morosan-Puopolo, Beate Brand-Saberi

**Affiliations:** Department of Anatomy and Molecular Embryology, Institute of Anatomy, Medical Faculty, Ruhr University Bochum, 44801 Bochum, Germany

**Keywords:** prenatal stress, glucocorticoids, skin development, chicken embryo

## Abstract

**Simple Summary:**

This study examined how prenatal stress affects embryonic skin development. For this purpose, the model organism of the chicken embryo was used to inject the stress hormone corticosterone at an early embryonic stage. After a certain period of stress hormone exposure, macroscopic observations and tissue examinations were undertaken in order to pursue this research question. The investigations demonstrated that physiological skin development was significantly impaired by prenatal stress. This could be attributed to the fact that both cell-internal and -external components promoting cellular integrity were downregulated by the effects of stress hormones. In addition, it could be shown that the physiological cell proliferation was decreased due to prenatal stress exposure. Since artificially-produced stress hormones, so-called synthetic glucocorticoids, are also frequently used in everyday clinical practice, the authors suggest a constant reevaluation of glucocorticoid-associated treatment strategies on the basis of these results.

**Abstract:**

Prenatal stress exposure is considered a risk factor for developmental deficits and postnatal behavioral disorders. While the effect of glucocorticoid-associated prenatal stress exposure has been comprehensively studied in many organ systems, there is a lack of in-depth embryological investigations regarding the effects of stress on the integumentary system. To approach this, we employed the avian embryo as a model organism and investigated the effects of systemic pathologically-elevated glucocorticoid exposure on the development of the integumentary system. After standardized corticosterone injections on embryonic day 6, we compared the stress-exposed embryos with a control cohort, using histological and immunohistochemical analyses as well as in situ hybridization. The overarching developmental deficits observed in the stress-exposed embryos were reflected through downregulation of both vimentin as well as fibronectin. In addition, a deficient composition in the different skin layers became apparent, which could be linked to a reduced expression of Dermo-1 along with significantly reduced proliferation rates. An impairment of skin appendage formation could be demonstrated by diminished expression of Sonic hedgehog. These results contribute to a more profound understanding of prenatal stress causing severe deficits in the integumentary system of developing organisms.

## 1. Introduction

Prenatal stress impairs developmental processes in the vertebrate embryo; as well, it influences physiological functions and behaviors after birth [1,2,3].

When the maternal organism is stressed, the hypothalamic–pituitary–adrenal axis becomes activated and glucocorticoids—commonly known as stress hormones—are secreted into the circulatory system [4]. Previous evidence has shown that maternal cortisol plasma levels correlate with fetal cortisol plasma levels [5,6]. Therefore, the occurrence of elevated glucocorticoid levels in fetal circulatory systems is a common event.

Glucocorticoids are lipophilic steroid hormones, which employ their functions—apart from other non-genomic pathways—by passing through the cells’ membranes and binding to the glucocorticoid receptor (GR) [7].

It has been observed that almost every organ system, such as the cardiovascular system, the immune system, the endocrine system, and the nervous system, can be influenced by stress-induced elevated glucocorticoid exposure [8,9,10,11].

Moreover, it is well-explored that glucocorticoids influence embryonic development in various ways [12]. By altering developmental mechanisms from “tissue accretion to differentiation” [12], the development of the brain, the heart, and skeletal muscle can be altered [13,14,15].

While numerous glucocorticoid-associated effects have been explored, their impact on the development of the skin, as the human body’s largest organ, is still partially unclear.

The skin and its appendages—also called the integumentary system—serve several essential functions. As the outermost layer of an organism’s body, the skin protects against environmental factors such as temperature and UV rays, it is part of the innate immune system by protecting against infectious organisms, it protects the body from dehydration, and it synthesizes vitamin D [16].

The skin of vertebrates is made of two major tissues: the epidermis and the dermis. The epidermis is the superficial layer, consisting of stratified squamous epithelial cells, which form the first barrier to the outer environment. It also contains immune cells, pigmented melanocytes, and cells that register tactile information. The dermis is separated from the epidermis by a basement membrane, and connects the skin to the subcutaneous tissues. It contains connective tissue with fibroblasts that produce an extracellular matrix comprising collagen and elastic fibers. The dermis also contains mechanoreceptors and cutaneous appendages, contributing to its numerous functions.

The effects of glucocorticoids on adult skin are well-explored. With their anti-inflammatory and immune-suppressive functions, they serve—in a dose-dependent manner—as therapeutics for various dermatological pathologies, such as different forms of eczema and psoriasis vulgaris [17]. Exemplary cytokines or transcription factors modulated by or within the steroid pathway are Interleukins, tumor necrosis factor-α (TNF-α), and nuclear factor ‘kappa-light-chain-enhancer’ of activated B-cells (NF-κB) [18,19].

One of the most common side effects of dermal glucocorticoid therapy is skin atrophy, presenting with skin hypoplasia and dysfunction, loss of elasticity, and an increase in fragility [17]. On a cellular level, glucocorticoids employ their negative side effects on the skin by inhibiting fibroblast proliferation and migration, and, moreover, altering the extracellular matrix [20,21,22]. The stress hormones reduce collagen synthesis and thereby also impair the process of dermal wound healing [23,24]. Furthermore, glucocorticoids have been determined to regulate apoptosis in keratinocytes [25].

Further investigation of the influence of prenatal stress exposure on skin development is of substantial interest because a viable skin is a crucial aspect of a physiologically-functioning and healthy organism.

The embryonic development of the skin is a complex process. While the origin of the epidermis is the ectoderm, the outermost layer of the three embryonic germ layers, the origin of the dermal connective tissue differs depending on the anatomic region. The dermis of the face and head develops mostly from neural crest cells, a temporary group of unique cells that arise from the ectoderm layer [26]. In the trunk region, the origin of the dermis can be divided into two groups: the dorsal dense dermis develops from the dermomyotome of the paraxial mesoderm, the so-called somites [27]; the dermis of the ventro-lateral body wall develops from the lateral plate mesoderm [28,29,30]. On embryonic day 6, the dermis is firstly distinguishable from the subcutaneous mesenchyme as a layer with higher cell density [31,32]. This differentiating dermal layer is characterized by Dermo-1 expression, which also plays a role during skin appendage development [33,34].

In the present study, we explored the influence of prenatal stress on the development of the skin in the avian embryo. Therefore, we investigated transcriptional changes of Dermo-1 after systemic glucocorticoid exposure during embryonic development. Moreover, we analyzed the expression of several other markers in the skin. For mesenchymal cells, we examined the expression of vimentin as an intermediate filament protein, which is responsible for strength and integrity of respective cells [35]. Moreover, we compared the expression of fibronectin, a protein that mediates interactions between mesenchymal cells and the extracellular matrix, regulating cell adhesion, migration, and differentiation [36]. To examine changes in cell proliferation, we compared the expression of phospho-histone H3 and proliferating cell nuclear antigen (PCNA) [37,38]. For specification of skin appendage development, we used Sonic hedgehog (SHH) as a relevant marker for epithelial placode formation during avian feather bud morphogenesis [39,40]. Lastly, as another epithelial marker important for epidermal maintenance and skin appendage growth, we investigated E-cadherin (E-Cad), a protein mediating intercellular adhesion [41].

Based on the summarized findings, we hypothesized that pathologically-elevated systemic prenatal stress exposure alters skin morphogenesis in the developing embryo. To investigate the hypothesis with the best possible comparability of glucocorticoid concentrations, the avian embryo is particularly suitable, because it allows for controlled administration of glucocorticoids with further analysis [42,43,44].

We performed in ovo injections of corticosterone (cort), a major avian physiological glucocorticoid, into the yolk sac of the developing chicken embryo, leading to an excessive systemic administration of the stress hormone during development [45]. Since the yolk sac stores the nutrients of the developing chicken embryo, the great advantage is that substances injected here gradually enter the circulation in a time-dependent manner. It should be mentioned here that the effects of the deliberately unphysiologically-high corticosterone concentration chosen may differ from chronic and low stress exposure in the developing organism. In order to select a reasonable and possibly early time point for the induction of glucocorticoid exposure, we relied on the results of a glucocorticoid expression analysis in chicken embryo, which revealed that—in the integumentary system—the associated glucocorticoid receptors are expressed from day 6 of development [46].

The knowledge gained by comparing the cort-exposed group with a control group contributes to a better comprehension of prenatal stress and its effects on embryonic development. Moreover, a deepened understanding of glucocorticoid signaling during vertebrate embryonic development is crucial for the discussion of exogenous glucocorticoid therapies in clinical contexts of prenatal medicine.

## 2. Materials and Methods

### 2.1. Chicken Embryo Treatment

Fertilized chicken eggs of *Gallus domesticus* were purchased from a local breeder, disinfected with 70% ethanol, and thereafter incubated at 37 °C and 80% relative humidity. The developmental stage was assessed according to Hamburger and Hamilton (HH) [47].

For our study, we used 140 fertilized chicken eggs, which were divided into a cort-exposed group (n = 70) and a control group (n = 70).

On the third day of embryonic development (E3) (Stages HH20-HH23), 3 mL of albumen was removed with a syringe, and a 2 cm-wide window was cut into the eggshell in order to access and inspect the embryo. Afterwards, the opened eggshell was covered with medical tape.

On embryonic day 6 (E6) of incubation (Stages HH28-HH30), 15 µg of corticosterone dissolved in 100 µL phosphate-buffered saline (PBS) with 1% ethanol was injected into the yolk sac of the developing chicken embryo, leading to a systemic administration of the glucocorticoid. For the experimental control, only the solvent of corticosterone—i.e., 100 µL PBS with 1% ethanol—was injected. The corticosterone dose used in this respective research design was based on previous investigations by Heiblum et al. (2001) [48], and additional dose-ranging experiments aimed at mimicking pathologically-elevated glucocorticoid concentrations.

The embryos were fixed after 2–7 days of further incubation by opening the amnion with forceps, and immersing them in 4% phosphate-buffered paraformaldehyde (PFA). After 24 h of fixation, the two experimental groups were compared.

### 2.2. Histological Analysis

The PFA-fixed specimens were washed in PBS and thereafter immersed in dehydrating ethanol solutions. After paraffin embedding, the embryos were sectioned transversally at 7 µm thickness. RotiHistol (Carl Roth, Karlsruhe, Germany) was used to remove the paraffin, and the rehydrated sections were treated with standard staining techniques.

The hematoxylin–eosin staining included immersing the sections in hematoxylin for 15 min, and afterwards in eosin for 2 min.

To differentiate the connective tissue of the skin, Masson–Goldner–Trichrome staining (Carl Roth, Karlsruhe, Germany) was performed. For this, the nuclei were stained with hematoxylin, according to Weigert, for 5 min. Afterwards, the trichromatic stain was conducted with ponceau–acid fuchsin, phosphotungstic acid–orange G, and 0.2% light green. A solution of 1% acetic acid was utilized for differentiation.

When the staining protocols were completed, the sections were dehydrated with ethanol solutions again, and covered with cover slips. For microscopic evaluation, the virtual slide microscope VS120 (Olympus, Tokyo, Japan)was utilized. Further analyses were carried out with Olympus OlyVia (Version 2.9) software and QuPath (Version 0.3.2) [49].

### 2.3. Immunohistochemical Analysis

The histological sections were deparaffinized and rehydrated as described above. Afterwards, they were put into a citrate buffer, which was microwaved in order to unmask antigens. After further washing with PBS, permeabilization with 1% Triton X 100 (Sigma Aldrich, St. Louis, MO, USA) and blocking with 7.5% bovine serum in PBS, the sections were treated with primary antibodies.

Vimentin (AMF-17b, Hybridoma Bank, Iowa City, IA, USA), fibronectin (B3/D6, Hybridoma Bank), and phospho-histone H3 (pHH3) (06-570, Merck, Darmstadt, Germany) were diluted in the blocking solution. Afterwards, the sections were incubated with the primary antibody solution overnight. The next day, the secondary fluorescent antibodies Alexa Fluor goat anti-mouse 488 and Alexa Fluor goat anti-mouse 568 (Thermo Fisher, Waltham, MA, USA) were pipetted onto the sections after removing the primary antibody and rinsing with PBS. 4′,6-Diamidino-2-phenylindol (DAPI) (Carl Roth, Karlsruhe, Germany) was utilized for staining the nuclei.

### 2.4. Whole-Mount In Situ Hybridization (ISH)

Whole-mount in situ hybridization was performed as previously described [50,51], using riboprobes for E-cadherin, Sonic hedgehog, Dermo-1, and proliferating cell nuclear antigen (PCNA). Proteinase K (10 μg/mL) (Sigma Aldrich, St. Louis, MO, USA) was applied onto the embryos for 20–40 min at room temperature to permeabilize the tissue. Thereafter, 1–2 μg/μL of the respective riboprobe was dissolved in a hybridization solution and applied onto the embryo for 48 h at 65 °C. The detection of the hybridization product was accomplished by an anti-DIG antibody conjugated to alkaline phosphatase (Roche, Basel, Switzerland).

After in situ hybridization, the specimens were photographed with a stereo microscope (M165 FC, Leica, Wetzlar, Germany) equipped with a digital camera (DFC420 C, Leica, Wetzlar, Germany). InkScape software was utilized for generating the figures (Version 1.2.1, 2022).

In order to generate sections of hybridized whole mounts, the embryos were embedded in 2.5–4% agarose gel and cut with a Vibratome (VT 1000 S, Leica, Wetzlar, Germany) at 50μm. The sections were collected with a brush, and covered with cover slips and Aquatex (Merck, Darmstadt, Germany). The sections were scanned and processed as described above.

### 2.5. TUNEL Analysis

The TUNEL-reaction was performed using a cell death detection kit (Elabscience, Wuhan, China). Briefly, the paraffin sections were deparaffinized and rehydrated as described above. Afterwards the TUNEL reaction was performed according to the manufacturer’s instructions.

DNAse was applied onto the section to serve as a positive control. For the negative control, the labeling was skipped. The control images are available as Figure S3 of the Supplementary Material.

### 2.6. RNA Isolation, Reverse Transcription, and Real-Time PCR (RT-PCR)

RNA isolation of the skin of chicken embryos was performed employing the TRI reagent (Sigma Aldrich, St. Louis, MO, USA). The cDNA was synthesized utilizing GoScript Reverse transcriptase (Promega, Madison, WI, USA). Afterwards, real-time quantification was conducted with GoTaq qPCR master mix (Promega, Madison, WI, USA). All steps were completed according to the respective manufacturer’s instructions. For the quantification of relative RNA levels, the Livak method was performed [52]. The displayed gene expression was normalized to 18 s. The primers used are available in Table S1 of the Supplementary Material.

### 2.7. Statistical Analysis

The Shapiro–Wilk test was employed in order to differentiate parametric from non-parametric distributions (*p* < 0.05). Following this evaluation, either an unpaired *t*-test or the Mann–Whitney U test was performed to assess significance. Significance is indicated by (*) for *p* < 0.05, (**) for *p* < 0.01, and (***) for *p* < 0.001. All results are presented as mean ± standard error.

## 3. Results

Apart from specific effects on skin development, systemic developmental effects were observed in the cort-exposed embryos including effects on extra-embryonic structures.

### 3.1. Prenatal Stress Impairs Angiogenesis of the Chorioallantoic Membrane (CAM)

Following prenatal in ovo corticosterone administration on embryonic day 6 (E6), the further development of chicken embryos was analyzed.

On E12—6 days after systemic prenatal stress exposure—the chorioallantoic membrane (CAM) showed a decrease in vascularization (Supplementary Material, Figure S1A,B). The number of blood vessels and moreover the diameter of the blood vessels were immensely reduced. The reduction of CAM-vascularization in the cort-exposed group in comparison to the control group was observed in 90% of cort-exposed embryos (control: n = 20; cort: n = 20).

### 3.2. Prenatal Stress Increases the Risk of Developmental Deficits

**Figure 1:**
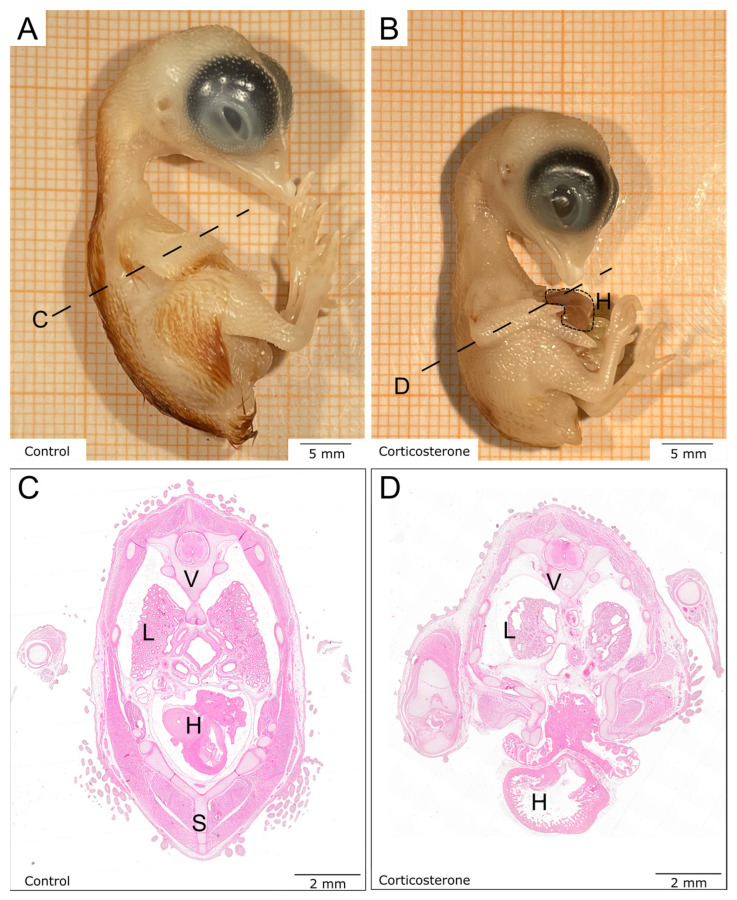
Prenatal stress exposure impairs overall development of avian embryos. The macroscopic images show lateral views of chicken embryos on E13. The control embryo (**A**) is further developed than the much smaller cort-exposed embryo (**B**). The open chest wall with the protruding heart is marked by a dotted line in (**B**). The transversal lines indicate the sectioning level for the histological sections (**C**,**D**). The control section (**C**) shows larger organs and a regular anatomy in comparison with the cort-exposed embryo section (**D**), which presents with an open chest wall and reduced organ size. H: heart, L: lungs, S: sternum, V: vertebrae.

On E13—7 days after systemic prenatal stress exposure—the developmental status of both experimental groups was compared. The survival rate on E13 of both groups was 75% (control: n = 20; cort: n = 20). Macroscopic evaluation of respective embryos showed a reduction in body size for the cort-exposed embryos (Figure 1A,B). Furthermore, the cort-exposed embryos presented developmental deficits and malformations such as ectopia cordis. The chest wall of cort-exposed embryos had not closed but remained with a bifid sternum, causing the heart to protrude through the chest wall (Figure 1C,D). Moreover, cort-exposed embryos showed less-developed eyes and smaller lungs. The musculoskeletal system was considerably less formed. For the skin appendages as part of the integumentary system, a reduction in the number of feather buds, as well as length and pigmentation, was observed (Figure 1A–D).

### 3.3. Prenatal Stress Reduces Embryonic Growth

Following macroscopic evaluation on E13, the embryos of both experimental groups were weighed and measured for growth analysis (Supplementary Material, Figure S2A,B).

For body length, a reduction was measured for the cort-exposed embryos. The mean body length of control embryos was 4.05 cm, while the mean body length of cort-exposed embryos was 2.88 cm. This is a significant decrease by 28.9% (*p* < 0.001; control: n = 20; cort: n = 20).

For bodyweight, a decrease was detected for the cort-exposed embryos. The mean bodyweight of control embryos was 3.97 g, while the mean bodyweight of cort-exposed embryos was 2.65 g. This is a significant decrease by 33.3% (*p* < 0.001; control: n = 20; cort: n = 20).

### 3.4. Prenatal Stress Reduces the Expression of Dermo-1

For Dermo-1, a gene encoding a transcription factor important for skin development and feather bud formation, a decrease in expression was observed.

**Figure 2:**
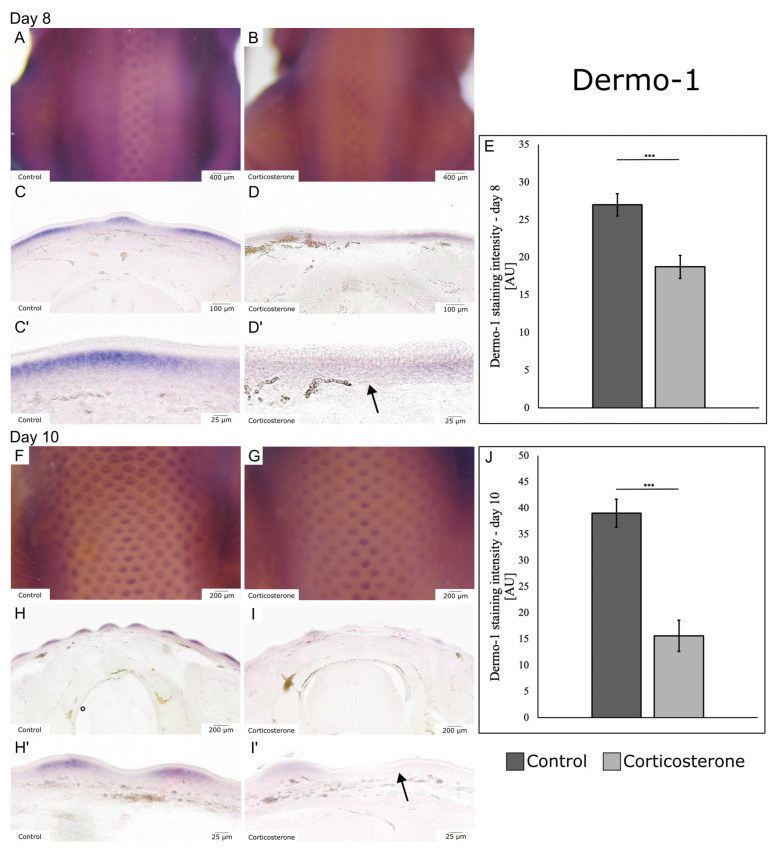
Prenatal stress reduces the expression of Dermo-1. The images show the expression patterns of Dermo-1 visualized by whole mount in situ hybridization and respective vibratome sections after systemic prenatal stress exposure. (**A**) shows a dorsal view of a hybridized control embryo on E8. (**B**) shows a dorsal view of a hybridized cort-exposed embryo on E8. Note the reduction in the number of feather buds. (**C**) shows a transversal vibratome section of (**A**). (**D**) shows a transversal vibratome section of (**B**). (**C**’) shows (**C**) in higher magnification. (**D**’) shows (**D**) in higher magnification. The quantity of transcripts ((**D’**), arrow), as well as the expression pattern of Dermo-1, was disturbed in cort-exposed embryos on E8. (**E**) displays the quantification of Dermo-1 expression on E8. The data are presented as mean ± standard error (control: n = 8; cort: n = 8). (**F**) shows a dorsal view of a hybridized control embryo on E10. (**G**) shows a dorsal view of a hybridized cort-exposed embryo on E10. (**H**) shows a transversal vibratome section of (**F**). (**I**) shows a transversal vibratome section of (**G**). (**H**’) shows (**H**) in higher magnification. (**I**’) shows (**I**) in higher magnification. On E10, cort-exposed embryos showed a reduction in transcripts of Dermo-1 ((**I**’), arrow) and a reduced number of developed feather buds. (**J**) displays the quantification of Dermo-1 expression on E10. The data are presented as mean ± standard error (control: n = 5; cort: n = 5). *** *p* < 0.001.

On E8—2 days after systemic prenatal stress exposure—the expression pattern of Dermo-1 was disturbed in cort-exposed embryos (Figure 2A–E). The control embryos showed extensive transcripts of Dermo-1 in the dorsolateral skin. Moreover, the first row of developing feather buds in the median line on the back of control embryos was positive for Dermo-1 (Figure 2A). The cort-exposed embryos showed an overall weaker expression of Dermo-1 in the dorsal skin, and a reduction in quantity of Dermo-1-positive feather buds (Figure 2B). The vibratome sections further showed that Dermo-1 expression was reduced in cort-exposed embryos (Figure 2C,D’, arrow). The quantification confirmed a significant decrease in Dermo-1 expression, indicated through a reduced ISH staining intensity by 30.5% in the cort-exposed group (Figure 2E; *p* < 0.001; control: n = 8; cort: n = 8).

On E10—4 days after systemic prenatal stress exposure—the expression pattern of Dermo-1 in cort-exposed embryos was further diminished. Dermo-1 transcripts were now mostly found in the feather buds of the dorsal skin (Figure 2F–I). Within one feather bud, the control embryos showed a cranio-caudal gradient of Dermo-1 transcripts, indicating a polarization in skin appendage establishment (Figure 2F). The cort-exposed embryos showed an immense decrease in Dermo-1 expression (Figure 2I’, arrow) and in the total feather bud number in comparison to the control group (Figure 2F–I). Moreover, the described polarization within individual feather buds was less prominent in cort-exposed embryos (Figure 2F,G). Furthermore, the vibratome sections revealed that the layer of Dermo-1-positive cells was thinner in cort-exposed embryos (Figure 2H,I). The quantification confirmed a significant decrease in Dermo-1 expression, indicated through a reduced ISH staining intensity by 60% in the cort-exposed group (Figure 2J; *p* < 0.001; control: n = 5; cort: n = 5).

### 3.5. Prenatal Stress Alters the Composition of the Different Skin Layers

To further investigate the effects of glucocorticoids on skin development, histological sections of E13 embryos—7 days after systemic prenatal stress exposure—from both experimental groups were analyzed and compared.

**Figure 3:**
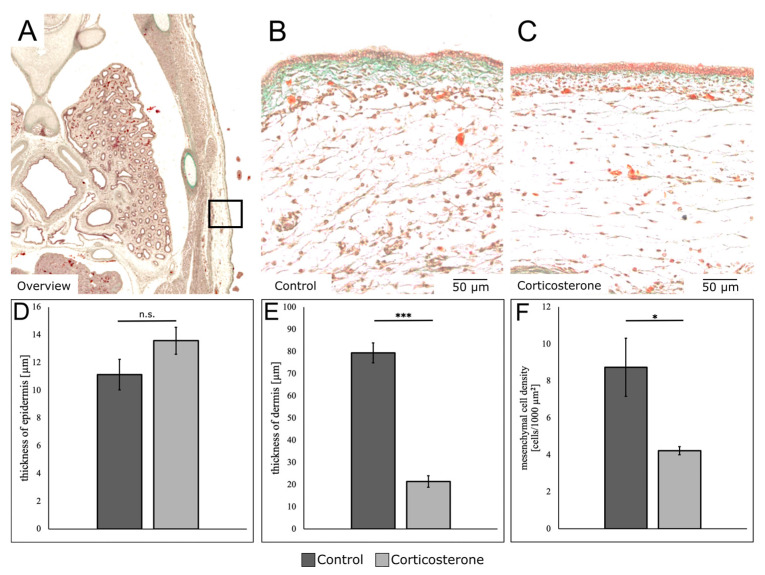
Prenatal stress alters the composition of the different skin layers. The images and diagrams show the influence of prenatal systemic stress exposure on the composition of the different skin layers. (**A**) shows an orientation overview of a transversal paraffine section stained with a Masson–Goldner–Trichrome staining in which the connective tissue appears green. The square indicates the region in the dorsolateral interlimb region from which the skin was analyzed and compared. (**B**) shows the skin of a control embryo. (**C**) shows the skin of a cort-exposed embryo. Note the reduction in dermis thickness and cell density for the cort-exposed embryo. The diagrams compare different parameters of skin composition such as the thickness of the epidermis (**D**), the thickness of the dermis (**E**), and the cell density in the subcutaneous mesenchymal layer (**F**). The data are presented as mean ± standard error (control: n = 5; cort: n = 5). * *p* < 0.05; *** *p* < 0.001; n.s.—not significant.

For the cort-exposed embryos, the sections revealed a minor increase in epidermis thickness. Moreover, the dermis—indicated by the green-appearing connective tissue—was remarkably reduced in size. The cell density in the subcutaneous mesenchyme was also visibly reduced (Figure 3B,C).

The histological sections were quantitatively compared. For the epidermis, we found an insignificant increase in thickness (21.9%) for the cort-exposed embryos (*p* = 0.08, control: n = 5; cort: n = 5) (Figure 3D). For the dermis, we found a significant decrease in thickness by 73% for the cort-exposed embryos (*p* < 0.001, control: n = 5; cort: n = 5) (Figure 3E).

Moreover, the cell density in the subcutaneous mesenchymal layer was significantly reduced by 51.2% (*p* = 0.032, control: n = 5; cort: n = 5) (Figure 3F).

### 3.6. Prenatal Stress Reduces the Expression of Mesenchymal Markers

To compare the expression of mesenchymal markers, we detected vimentin as an intracellular intermediate filament and fibronectin as a protein of the extracellular matrix, employing immunohistochemical methods, on E13—7 days after systemic prenatal stress exposure.

**Figure 4:**
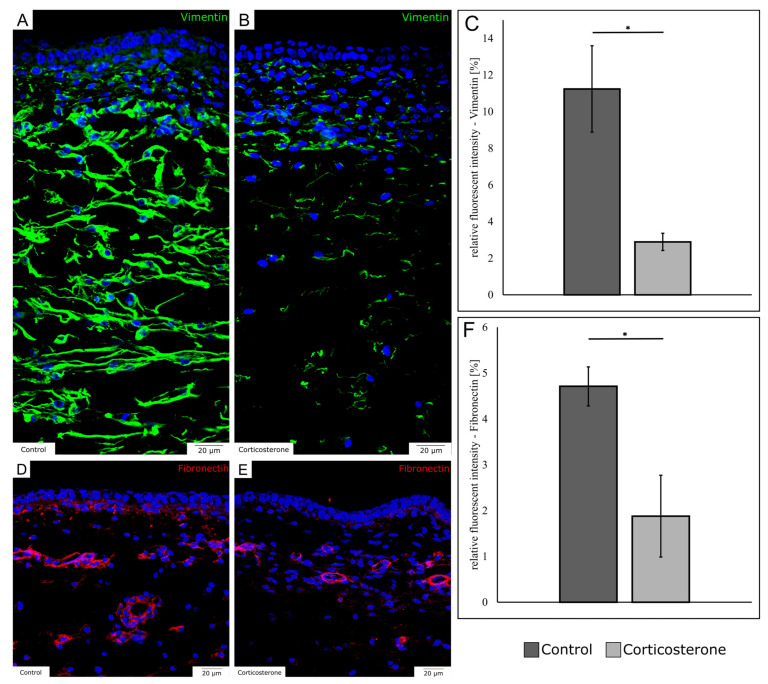
The images demonstrate the decrease in the expression of mesenchymal markers in the skin on E13. The images (**A**) (control) and (**B**) (corticosterone) show the immunohistochemical detection of vimentin. Note the reduction of vimentin expression in the dermis and subcutaneous mesenchyme. (**C**) shows the respective quantification of the fluorescent intensity. The images (**D**) (control) and (**E**) (corticosterone) show the immunohistochemical detection of fibronectin. Note the reduction of fibronectin expression in the dermis. (**F**) shows the respective quantification of the fluorescent intensity. The data are presented as mean ± standard error (control: n = 3; cort: n = 3). * *p* < 0.05.

For vimentin, the immunohistochemical staining showed a decrease in expression in the cort-exposed embryos, especially prominent in the subcutaneous mesenchyme (Figure 4A,B). The filaments appeared shorter and thinner, and the pattern was less organized in comparison to the control. The quantification of vimentin expression revealed a decrease in fluorescent intensity by 74.3% in cort-exposed embryos (Figure 4C) (*p* = 0.037; control: n = 3; cort: n = 3).

For fibronectin, the immunohistochemical sections demonstrated that fibronectin expression was remarkably reduced in the dermal connective tissue (Figure 4D,E). Moreover, a minor reduction of fibronectin expression was found in subcutaneous perivascular regions. The quantification showed that the relative fluorescent intensity of fibronectin was reduced by 60.1% in cort-exposed embryos (Figure 4F; *p* = 0.032; control: n = 3; cort: n = 3).

### 3.7. Prenatal Stress Reduces the Mitotic Activity in the Skin

**Figure 5:**
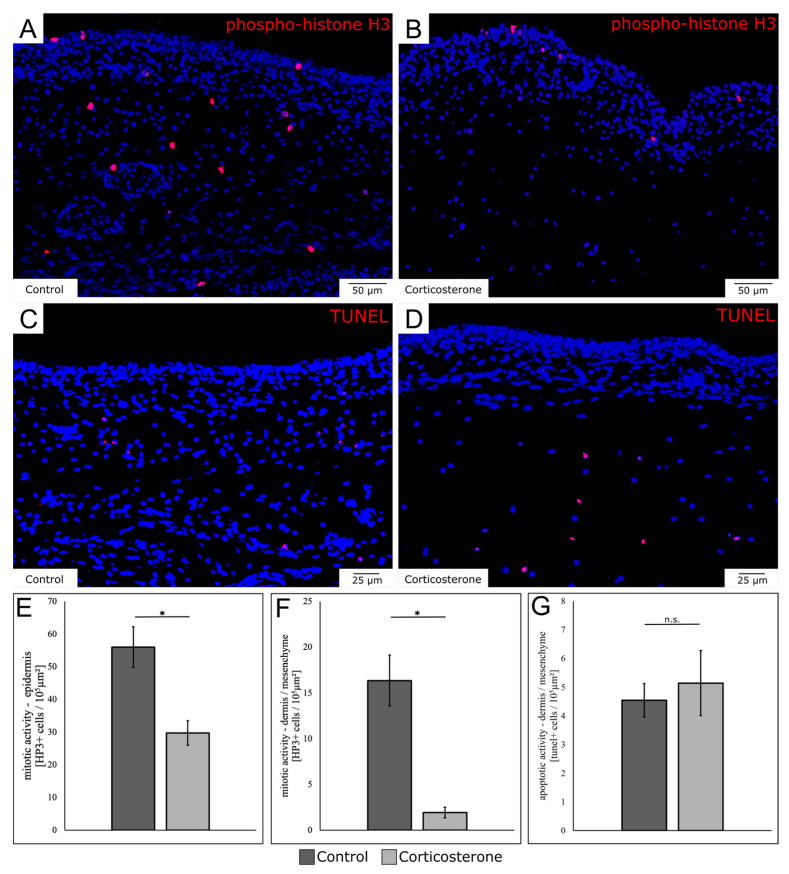
The images show the comparison of mitotic and apoptotic activity in the skin on E13 after prenatal systemic stress exposure. (**A**,**B**) show the immunohistochemical detection of phospho-histone H3, indicating cell proliferation. (**C**,**D**) show the TUNEL-detection visualization of apoptotic cells. The diagrams show the respective quantifications of proliferating phospho-histone H3-positive cells (phh3+ cells) for both the epidermis (**E**) and the dermis (**F**), as well as of the apoptotic TUNEL-positive cells in the dermis (**G**). The data are presented as mean ± standard error (control: n = 3; cort: n = 3). * *p* < 0.05; n.s. = not significant.

The mitotic activity on E13 in the skin was analyzed, using immunohistochemistry for phospho-histone H3 and quantifying the positively-stained cells (phh3+ cells) (Figure 5A,B). For the epidermis, the number of mitotic cells was significantly reduced in the cort-exposed group by 46.9% (Figure 5E; *p* = 0.03; control: n = 3; cort: n = 3).

For the dermis and the subdermal mesenchyme, the number of mitotic cells was significantly reduced by 88.4% in the cort-exposed embryo group (Figure 5F; *p* = 0.026; control: n = 3; cort: n = 3).

The apoptotic activity on E13 in the skin was assessed using a TUNEL-reaction (Figure 5C,D). While the number of positively-stained apoptotic cells (TUNEL+ cells) was elevated by 13% in the cort-exposed embryos, the difference was insignificant (Figure 5G; *p* = 0.364; control: n = 3; cort: n = 3). For positive and negative controls of the TUNEL-reaction, see Figure S3 of the Supplementary Material.

### 3.8. Prenatal Stress Impairs Embryonic Skin Appendage Formation

To analyze skin appendage formation, we performed whole-mount in situ hybridization (ISH) on E11—five days after systemic prenatal stress exposure—with different markers for feather establishment.

**Figure 6:**
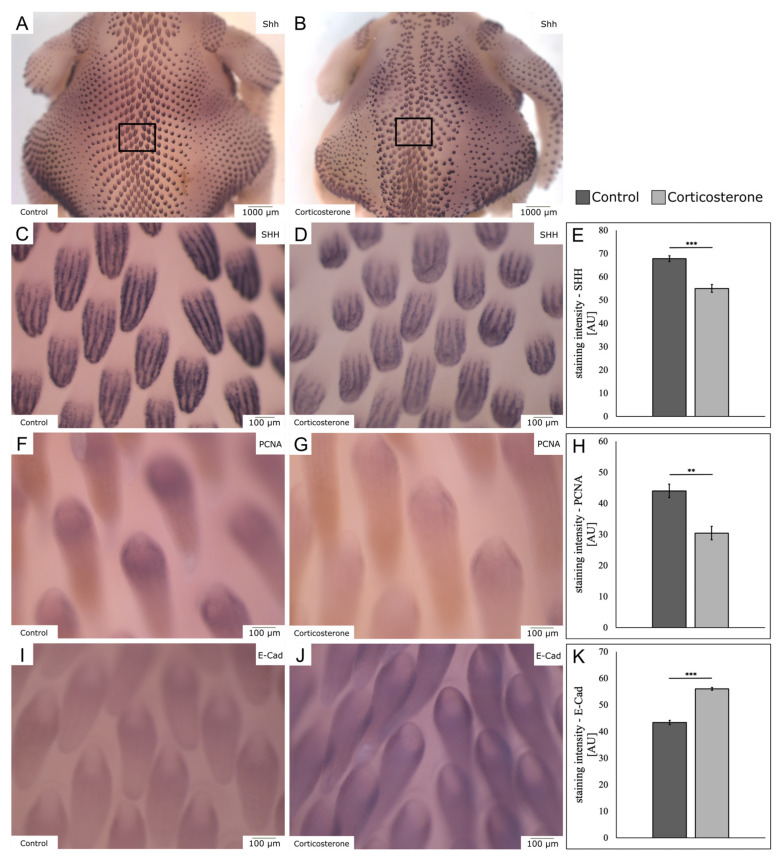
The images demonstrate skin appendage formation using different markers for whole-mount in situ hybridization on E11, 5 days after systemic stress exposure. (**A**,**B**) display an exemplary dorsal overview of Sonic hedgehog (SHH) for the control and cort-exposed embryos. The squares indicate the areas of further comparison. (**C**,**D**) compare the expression of SHH in higher magnification. (**E**) displays the respective quantification for SHH. (**F**,**G**) demonstrate expression of proliferating cell nuclear antigen (PCNA). (**H**) displays the respective quantification for PCNA. (**I**,**J**) show the expression of E-cadherin (E-Cad) for both the control and cort-exposed embryos. (**K**) displays the respective quantification for E-Cad. The data are presented as mean ± standard error (control: n = 5; cort: n = 5). ** *p* < 0.01; *** *p* < 0.001.

For Sonic hedgehog (SHH), we found a disorganized and asymmetric feather pattern, with fewer and less-developed feather buds on the dorsal skin of cort-exposed embryos (Figure 6A,B). The higher magnification shows that, in the cort-exposed group, the feather buds were smaller in size. Moreover, they showed a different, rounder shape and fewer transcripts of SHH (Figure 6C,D), indicated by a significant decrease by 18.9% in ISH staining intensity in the cort-exposed embryos (Figure 6E; *p* < 0.001; control: n = 5; cort: n = 5).

For proliferating nuclear cell antigen (PCNA), a further mitotic marker, we found a decrease in mRNA expression indicated through a reduced ISH staining intensity by 30.9% in the cort-exposed embryo group (Figure 6F–H; *p* = 0.001; control: n = 5; cort: n = 5).

The expression of E-cadherin (E-Cad), an epithelial adhesion protein, was upregulated in the feather buds of the cort-exposed embryos, indicated through an increased ISH staining intensity by 29% in this group (Figure 6I–K; *p* < 0.001; control: n = 5; cort: n = 5).

### 3.9. Prenatal Stress Increases Expression of NF-κB in the Skin

For further comprehension of transcriptional changes within the steroid pathway, embryonic skin from both the control and the experimental group was isolated on E10—4 days after systemic prenatal stress exposure—and analyzed via RT-PCR.

**Figure 7:**
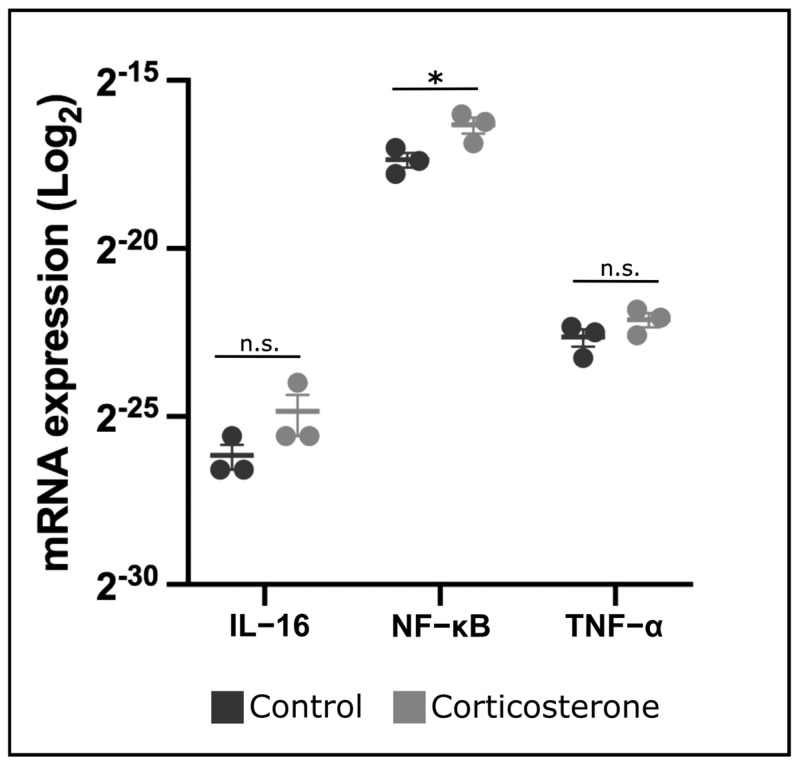
The diagram displays transcriptional changes within the steroid pathway in the skin of chicken embryos on E10—4 days after systemic prenatal stress exposure—analyzed via RT-PCR. The data are presented as mean ± standard error (control: n = 3; cort: n = 3). * *p* < 0.05; n.s. = not significant.

For nuclear factor ‘kappa-light-chain-enhancer’ of activated B-cells (NF-κB), we determined a significant increase by 51.2% in mRNA expression in the cort-exposed group (Figure 7; *p* = 0.033; control: n = 3; cort: n = 3).

For Interleukin-16 (IL-16) and tumor necrosis factor-α (TNF-α), the transcriptional changes between the control and the cort-exposed groups were insignificant (Figure 7; *p* = 0.141 and *p* = 0.095, respectively; control: n = 3; cort: n = 3).

## 4. Discussion

The goal of the presented research was to analyze the effects of prenatal stress on avian embryonic development, with a focus on the integumentary system. Following in- ovo injections of corticosterone, we observed stress-induced developmental deficits, and, moreover, a disturbance in skin establishment and skin appendage formation through histological and molecular biological methods.

Embryos from the cort-exposed group presented an impairment in the formation of the chorioallantoic membrane, overall retardation in development indicated through a reduction in body growth, an increased incidence of chest wall malformations, and decreased organ size. The observations of developmental deficits and growth retardation in prenatally-stressed organisms is congruent with numerous previous findings [53]. The prenatal administration of the synthetic glucocorticoid dexamethasone has been previously-described to cause growth retardations in avian embryos [53]. Moreover, these findings are compliant with mammalian model organisms such as rats, which displayed intrauterine growth retardation after being exposed to prenatal stress [53]. This supports the idea that the observed effects of glucocorticoids are comparable across species and across different kinds of glucocorticoids.

For the chorioallantoic membrane (CAM), we detected an impaired vascularization in the cort-exposed group. This is in line with various previous descriptions, elaborating on the angiostatic potential of glucocorticoids [54]. The resulting decrease in CAM vascularization—possibly leading to hypoxic conditions—could be a contributing factor in the developmental global and skin-related deficits observed in the cort-exposed group, with potentially long-lasting consequences into adulthood [55,56].

Our findings of a higher incidence in anterior body wall malformations in the cort-exposed group are in line with observations by Xu et al. (2021), who detected skeletal muscle abnormalities and ventral body wall deformities in chicken embryos [57] that had been exposed to the synthetic glucocorticoid dexamethasone in an earlier stage than in our experimental design.

For avian skin development, the gene Dermo-1 has been shown to play an important role in dermis differentiation and in initiating skin appendage formation [34,58]. Previous investigations revealed that an overexpression of Dermo-1 in avian embryos results in a feather-tract typical increase in dermis thickness and higher cell density of the dermal mesenchyme, acting as an early positive regulator of skin appendage establishment upstream from epidermal β-catenin and dermal FGF-10 [58]. Our results of a corticosterone-induced decrease in Dermo-1 expression, accompanied by a reduction in dermis thickness, further contextualize these previous findings, particularly because a forced Dermo-1 upregulation has been shown to provoke a denser and more thickened dermis in avian embryos [58]. It is important to note that Dermo-1 knockout in zebrafish did not prevent skin development completely, but resulted in a “qualitative change” [59] in skin appendage formation, further suggesting a functional role of Dermo-1 in the morphology and organization of cutaneous appendages [59]. Our findings of a disturbed feather bud pattern after glucocorticoid-induced downregulation of Dermo-1 in the avian embryo supports this concept.

Furthermore, we demonstrated that the impairment of embryonic dermis formation by prenatal stress hormones is also comparable with the described effects of glucocorticoids on adult skin and connective tissue. Glucocorticoids are known to cause skin atrophy resulting in morphological changes of the dermis, presenting an inhibition of fibroblast proliferation and reduced collagen synthesis [17,60,61]. Our findings of a decrease in mitotic activity, cell density, and dermis thickness further extend these observations for the avian embryonic organism. The negative effects of a systemic glucocorticoid exposure on the subdermal mesenchyme, indicated through a downregulation of vimentin and fibronectin, contextualize and strengthen previous observations suggesting that glucocorticoids induce mesenchymal-to-epithelial transition via inhibiting TGF-β1-signaling [62,63]. This is further supported by the upregulation of the epithelial adhesion protein E-cadherin, as well as the comparably smaller decrease in epidermal cell proliferation in the cort-exposed group. Moreover, this is consistent with observations in glucocorticoid receptor (GR) overexpressing mice, which, accordingly, presented epidermal hypoplasia [64].

Taken together, these processes could hypothetically be explained by the concept suggested by Fowden et al. (2015), who stated that prenatal glucocorticoid exposure induces a switch from tissue growth towards tissue differentiation [12]. This includes a decrease in cell number in certain tissues, but, moreover, an activation of certain cell differentiation pathways, and a shift of proliferation towards tissues that are more likely to ensure neonatal viability [12]. Hypothetically, the upregulation of the intercellular adhesion protein E-cadherin could contribute to the environmentally stressor-adapted phenotype of the developing organism.

For the skin appendages, we found underdeveloped feathers with a more disorganized feather bud distribution in the cort-exposed group. Moreover, we detected a decrease in SHH expression in the developing appendages, indicating an altered formation of the epithelial placodes [39,40]. The observation of a glucocorticoid-induced downregulation of SHH in the developing integumentary system is in line with the findings of decreased SHH expression during avian somitic differentiation after earlier dexamethasone administration [57]. The reduced mitotic activity in the skin, indicated trough a decrease in PCNA expression and in the number of phospho-histone H3-positive cells, could be a contributing factor towards the underdevelopment of the feathers, and is congruent with previous descriptions of a glucocorticoid-induced inhibition of cellular proliferation [65,66].

Regarding the modulation of potential mediators within the steroid pathway, we detected an increase in the expression of NF-κB in the skin of stress-exposed embryos. NF-κB has been characterized as an important transcription factor in skin morphogenesis and homeostasis [67,68]. While therapeutic glucocorticoids are commonly-known for their anti-inflammatory capacity—associated with a downregulation of inflammatory cytokines and transcription factors such as NF-κB [19]—prenatal maternal stress has been described to cause an increase in the inflammatory response of developing organisms [69,70]. Moreover, prolonged GR activation—as is the case in chronic stress or in the present developmental model—has been shown to cause synergistic effects between GR and NF-κB [71,72], supporting the notion that the detrimental developmental deficits seen after prenatal systemic corticosterone administration are mediated through glucocorticoid signaling.

## 5. Conclusions

Systemic prenatal stress exposure impaired the overall developmental status of avian embryos. Glucocorticoid administration led to decreased vascularization of the CAM, reduced body growth, and malformations of the ventral body wall.

Focusing on the integumentary system, we determined a reduced Dermo-1 expression, resulting in an altered composition of the different skin layers, with a decrease in dermis thickness and, moreover, a disturbance of the subdermal mesenchyme, indicated by a reduced expression of mesenchymal markers, cell density, and cellular proliferation. Moreover, the formation of skin appendages was impacted, shown by a reduced expression of SHH and PCNA, as well as an increase in E-cadherin expression. However, because of the deliberately unphysiologically-high corticosterone concentration chosen, the results may not be transferable to physiologically slightly-elevated corticosterone concentrations.

The avian embryo serves as a useful model organism for prenatal stress research, allowing for a controlled administration of glucocorticoids. The insights gained contribute to a better comprehension of the effects of prenatal stress on vertebrate embryonic development, and the role of glucocorticoid exposure during the developmental establishment of the integumentary system.

## Data Availability

Data are contained within the article.

## Supplementary Materials

The following supporting information can be downloaded at: https://www.mdpi.com/article/10.3390/biology12050656/s1, Figure S1: vascularization of the CAM in cort-exposed embryos; Figure S2: body length and weight of cort-exposed embryos; Figure S3: controls of the TUNEL-reaction; Table S1: primers used for RT-PCR.
