# Supplementary material for: Systemic Prenatal Stress Exposure through Corticosterone Application Adversely Affects Avian Embryonic Skin Development
Source: Biology (Basel). 2023 Apr 26;12(5):656. doi: 10.3390/biology12050656 (PMC10215502; doi:10.3390/biology12050656)
Supplement: Supplementary file 1 [file biology-12-00656-s001.zip › biology-2190072-supplementary.pdf]

Supplementary Materials

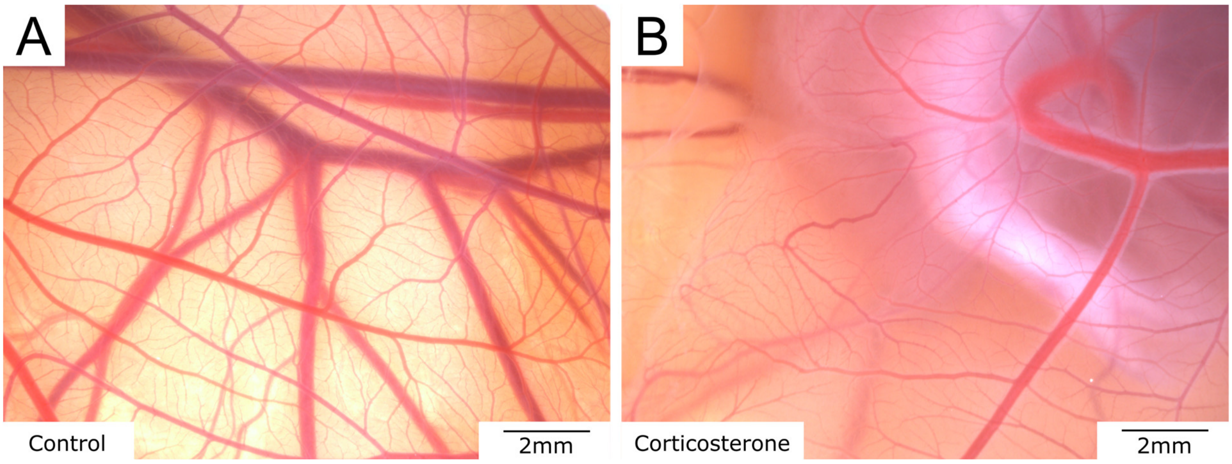

Supplementary Figure S1. Prenatal stress exposure impairs vascularization of the avian embryonic CAM. The vascularization of the CAM on E12 is shown in ovo for the control (**A**) and for a corticosterone-exposed embryo (**B**). Note the reduction of blood vessel quantity and diameter.

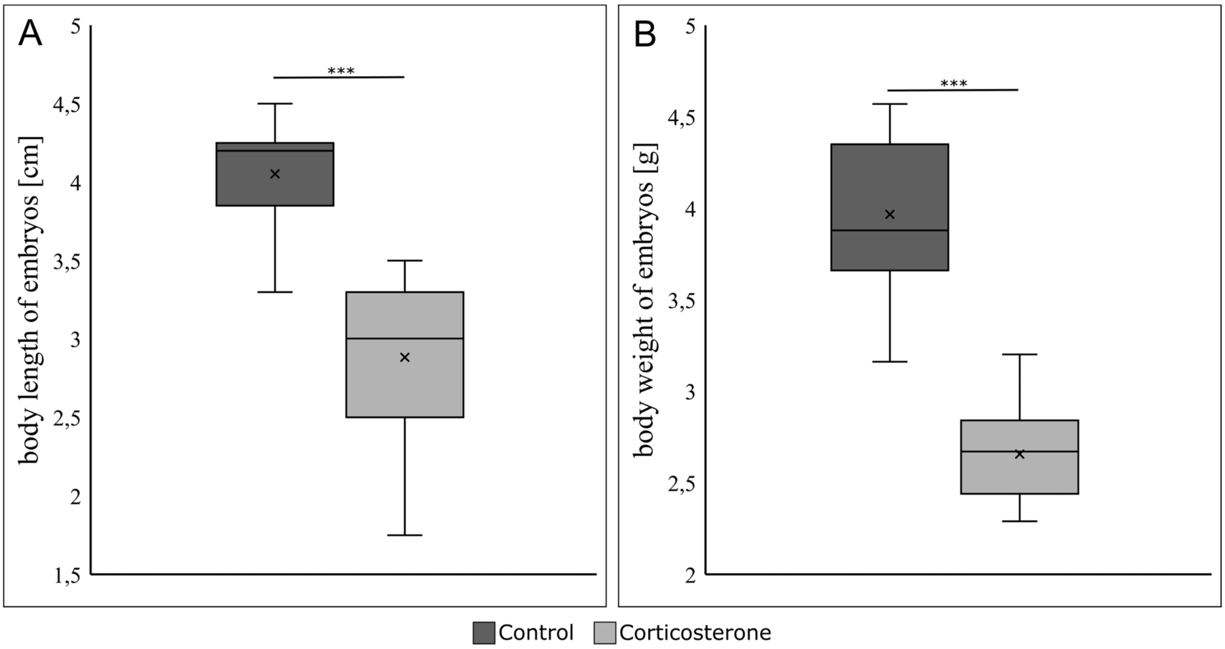

Supplementary Figure S2. The box plots show the distribution of body length (**A**) and body weight (**B**) across the control and cort-exposed embryos on E13. Both body length and body weight were significantly reduced in cort-exposed embryos (control: n=20; cort: n=20) *** $p<0{,}01$.

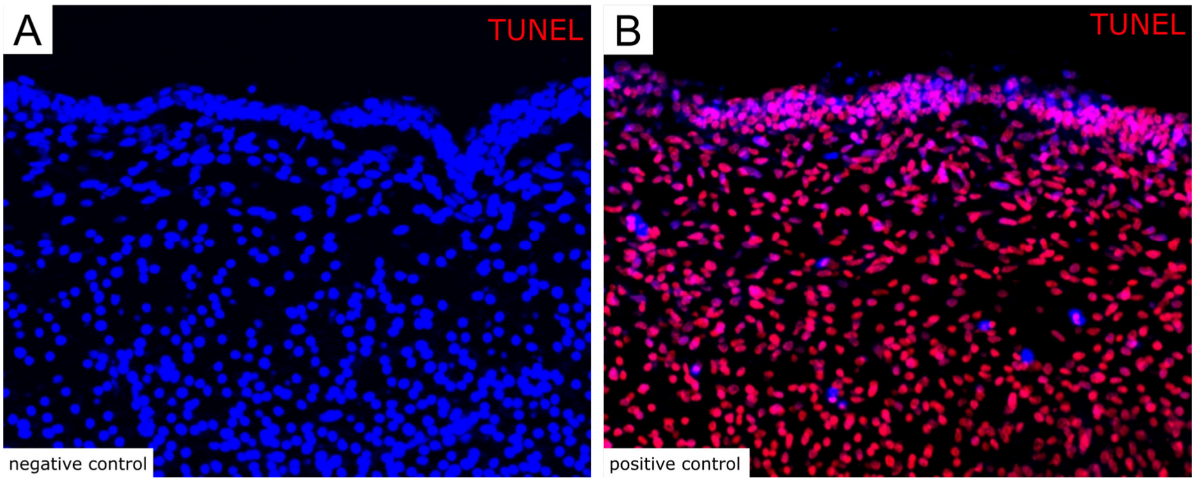

Supplementary Figure S3. The images display the positive (**A**) as well as the negative (**B**) controls of the TUNEL-reaction on paraffin sections of the skin of chicken embryos on E13.

Supplementary Table S1. The table displays the primers used for RT-PCR.

Gene	Primer sequence
NF-κB (nuclear factor ‘kappa-light-chain-enhancer’ of activated B-cells)	Forward: 5′ CAGTCAACGCAGGACCTAAA 3′<br>Reverse: 5′ GTTAGCTGTCTGTCTCCACATC 3′
TNF-α (tumor necrosis factor-α)	Forward: 5′ GACAGCCTATGCCAACAAGTA 3′<br>Reverse: 5′ GAATTAAGCAACAACCAGCTATG 3′
IL-16 (Interleukin-16)	Forward: 5′ GCGAGAACAGCATGGAGATG3 ′<br>Reverse: 5′ GTAGGTCTGAAAGGCGAACAG 3′
